# Acute fatiguing effects and biomechanical characteristics of a single bout eccentric quasi-isometric resistance exercise of ankle plantar flexors

**DOI:** 10.3389/fbioe.2025.1517098

**Published:** 2025-06-25

**Authors:** Luka Križaj, Nejc Šarabon, Žiga Kozinc

**Affiliations:** ^1^ Faculty of Health Sciences, University of Primorska, Izola, Slovenia; ^2^ Andrej Marušič Institute, University of Primorska, Koper, Slovenia; ^3^ Ludwig Boltzmann Institute for Rehabilitation Research, St. Pölten, Austria

**Keywords:** eccentric quasi-isometric contraction, ankle plantar flexors, intensity, exercise, eccentric contraction

## Abstract

**Introduction:**

Investigation of the eccentric quasi-isometric (EQI) resistance exercise has started in recent years. However, the biomechanical characteristics and fatigue effects of EQI muscle actions in the ankle plantar flexors remain unexplored. This study aimed to compare the biomechanical characteristics of plantarflexion EQIs and post-EQI acute fatigue between two different loading intensities.

**Methods:**

Twenty regularly physically active participants (9 men, 11 women) completed 3 sets comprising of a single EQI muscle contraction at 75% and 90% (one intensity for each leg in a random order) of maximal voluntary isometric contraction (MVIC) on an isokinetic dynamometer. Outcome variables included total contraction time, torque impulse, angular velocities and range of motion during EQI muscle actions, as well as pre- and post-contraction MVIC measures.

**Results:**

The analysis indicated no statistically significant effect of EQI muscle-action on MVIC torque (main effect of time: p = 0.636). There were also no statistically significant differences between EQI muscle actions performed at 75% and 90% MVIC regarding angular velocity (p = 0.244). However, executing EQI muscle contraction at 75% MVIC resulted in statistically significantly larger total contraction time, total torque impulse, and range of motion (p < 0.001) compared to EQI at 90% MVIC.

**Discussion:**

These findings suggest that performing EQI contractions at 75% MVIC may be more effective for generating greater mechanical stimulus without inducing significant acute fatigue.

## 1 Introduction

In recent years, many studies in the field of movement and exercise have focused on the effects of different forms of exercise, mainly focusing on the differences between the effects of varying exercise volume (Habets and van Cingel, 2015; Head et al., 2019), intensity (Luan et al., 2019; Oranchuk et al., 2019; Wiegerinck et al., 2013) and type of muscle contraction to optimise exercise (Henderson et al., 2023; Ličen et al., 2024; Oranchuk et al., 2019; Oranchuk et al., 2021; Oranchuk, et al., 2021). The latter is defined in terms of whether the exercise is based on either isometric contraction (a form of contraction characterized by no alteration in the length of the muscle-tendon complex) (Merza et al., 2021; Radovanović et al., 2022; Young and Press, 1994), concentric contraction, which involves a shortening of the muscle-tendon complex (Malliaras et al., 2013; Young and Press, 1994), eccentric contraction, characterized by lengthening of the muscle-tendon complex (Alfredson and Pietila, 1998; Rabello et al., 2020; Young and Press, 1994) or isotonic contraction, which includes both concentric and eccentric contractions (Grävare-Silbernagel et al., 2001; Murphy et al., 2019; Young and Press, 1994).

In recent years, eccentric quasi-isometric (EQI) resistance exercise has been suggested as a potentially effective approach for exercise optimisation (Oranchuk et al., 2019). This method permits the application of high loads without the risk of a sudden increase in mechanical stress on the tendon and joint (Arnoczky et al., 2007; Magnusson and Kjaer, 2019). EQI muscle contraction is defined as “holding the position until isometric failure, followed by maximum resistance in the following eccentric phase and an attempt to re-establish isometric contraction” (Oranchuk et al., 2019). EQI resistance exercise can be most accurately achieved on an electromechanical dynamometer, set to isotonic mode. The contraction begins by moving the joint to the position where the selected muscle group is most shortened (by concentrically contracting the muscle), followed by the attempt to isometrically maintain the joint position (Oranchuk et al., 2021; Oranchuk et al., 2021). As the muscle group fatigues under a constant load, it undergoes an eccentric contraction (Oranchuk et al., 2021; Oranchuk et al., 2021). The isometric and eccentric contraction phases alternate until the end of the predefined RoM of the selected joint (Oranchuk et al., 2021; Oranchuk et al., 2021).

Only a limited number of studies have been conducted to investigate the EQI paradigm, focusing on the knee extensors (Oranchuk et al., 2021; Oranchuk et al., 2021; Ličen et al., 2024), knee flexors (Ličen et al., 2024) and the elbow flexors (Henderson et al., 2023), respectively. Oranchuk et al. (2021) compared the EQI protocol with a contraction load set to 70% of maximal voluntary isometric contraction (MVIC) to an eccentric protocol with an equalized torque impulse. They reported that EQI protocol led to significantly less pronounced delayed onset muscle soreness, a smaller reduction in MVIC, and lower ultrasound echo intensity of the muscle compared to eccentric protocol (Oranchuk et al., 2021). Another study investigated four repetitions of EQI contractions at 70% MVIC with 3-min rests between the repetitions, reporting several joint-angle-specific differences in time-normalized and absolute variables (Oranchuk et al., 2021). In addition, significant differences (p < 0.001) in the total angular impulse were found between the first and the fourth repetition (Oranchuk et al., 2021). Meanwhile, Ličen et al. (2024) reported that performing four EQI contractions reduces the maximum and average knee extensor strength (p < 0.001; d = 0.70–0.71) and increases the optimum angle or the angle where the maximum torque was recorded during the stretch (p < 0.001; d = 1.00), measured before and after the four EQI protocol. In the case of knee flexors, only a decrease between the maximum and average torque was observed (p ≤ 0.022; d = 0.36), whereas no change in the optimum angle (p = 0.811; d = 0.06) was found between the initial and final measurement of the maximum torque after the four EKINs were performed (Ličen et al., 2024). Only one study focused on the effects of the four-repetitions EQI protocol on elbow extensors. Henderson et al. (2023) also found that within an EQI protocol with contraction at 50% MVIC, time under tension decreased with subsequent repetitions of the EQI contraction. They also found that men tended to display greater absolute strength (MVIC), external load, and contraction velocity compared to women; however, statistically significant sex differences were only observed for MVIC and external load, not for contraction velocity (Henderson et al., 2023). However, the time under tension of each of the four repetitions tended to last longer on average for women compared to men, but there were no statistically significant differences between mention variables.

Building on prior research on knee extensors (Oranchuk et al., 2021; Oranchuk et al., 2021), knee flexors (Ličen et al., 2024) and elbow flexors (Henderson et al., 2023), our study seeks to extend these investigations by examining the effects of EQI resistance exercise on the ankle plantar flexors at different loads. The specific objective of this study is to explore the biomechanical characteristics of EQI muscle actions, such as total contraction time, total torque impulse, mean angular velocity, and total RoM between 75% or 90% of MVIC intensities. The intensity levels were chosen based on the findings of other studies (Carvalho et al., 2022; Schoenfeld et al., 2017), which found that moderate-to-high loading (over 60% MVIC) had a greater effect on increasing muscle strength and hypertrophy than low loading. Additionally, other studies that focused on surface electromyography have also found that the mean electrical amplitude was lower with low-intensity exercise than with load exceeding 80% MVIC, even when sets were performed to muscle failure (Jenkins et al., 2015; Schoenfeld et al., 2014). Based on the above, we aimed to avoid lower loads in this study, which in the context of resistance training typically refers to intensities below 50%–60% of MVIC and may not effectively promote neuromuscular adaptations (Schoenfeld et al., 2017). Therefore, even though only a single bout of exercise was performed, we selected two intensities (75% and 90% of MVIC) that fall within the range considered appropriate for inducing meaningful neuromuscular adaptations. These values were also chosen to ensure a sufficiently large contrast between the two loading conditions. We hypothesized that lower intensity (75% MVIC) EQI exercise protocol will result in greater total torque impulse, total contraction time, and RoM compared to higher intensity (90% MVIC) EQI exercise. We also hypothesized that both intensities would result in a statistically significant MVIC decrease when comparing pre- to post-MVIC measurements. With this study, we aim to significantly expand the understanding of EQI muscle actions, particularly their biomechanical impacts on the ankle plantar flexors at varying loads.

## 2 Methods

### 2.1 Participants

For the study, 20 healthy young adults (11 women, 9 men) were recruited. Due to the novelty of directly comparing EQI contractions between different intensities and including a different muscle group, determining an appropriate sample size through conventional power analysis was challenging. The sample size was similar to previous studies with a similar design which focused on knee muscles (Oranchuk et al., 2021; Ličen et al., 2024). The participants were students recruited through social media posts and word of mouth. The inclusion criteria for the study were the absence of musculoskeletal injuries in the last 3 months, any ankle injuries in the past year, and at least a moderate experience with resistance training (minimal of average 2 sessions per week for the past 6 months). Exclusion criteria included pregnancy, the presence of chronic non-communicable diseases, and prior experience with EQI-based training. Participants were required to sign an informed consent form to participate in the research. The study was approved by the Commission of the Republic of Slovenia for Medical Ethics (No. 0120-690/2017/8).

### 2.2 Study design and procedures

The measurements took place between 19 February 2024 and 23 March 2024. Each subject completed the protocol in 1 day. This study employed a cross-sectional, within-participant design in which each participant’s two legs were utilized to examine the effects of moderate (75% MVIC) and high (90% MVIC) load EQI muscle actions The assignment of intensity to the left and right legs, as well as the sequence of measurements, was quasi-randomized using a Latin square design. The protocol involved participants first undertaking plantar flexion MVIC assessments, followed by three EQI muscle contraction with 2-min intervals for rest. Immediately after the final EQI set, another MVIC measurement was conducted to evaluate the fatiguing effects of the EQI protocol. Following a 5-min rest period, the entire procedure was replicated with the alternate leg. The full measurement protocol was developed jointly by the authors, while the measurements were carried out entirely jointly by LK and ŽK. All assessments were conducted in a consistent environment, specifically, in an air-conditioned room with the temperature maintained between 22°C and 23°C.

### 2.3 Maximal voluntary isometric contraction

**FIGURE 1:**
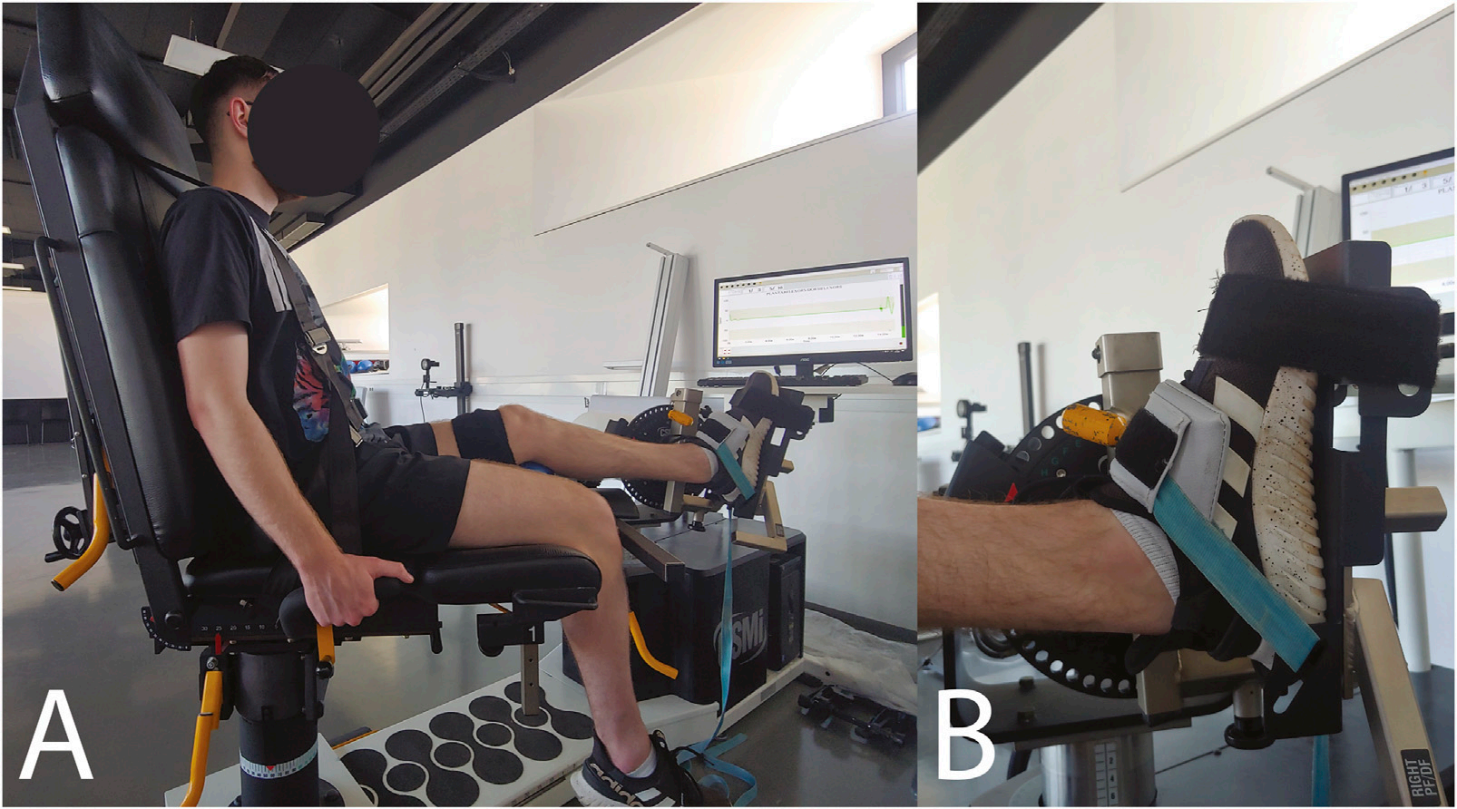
**(A)** Participant positioning and fixation in the dynamometer. **(B)** Close-up of ankle and foot fixation.

MVIC assessment was performed on an isokinetic dynamometer (HumacNorm, Computer Sports Medicine Inc., Massachusetts, United States). The dynamometer backrest inclination was set to 80° in all participants (Figure 1A). Although there was no additional lumbar inclination, we ensured that the lumbar region of all participants was supported by pads and was slightly curved to maintain the natural arch of the lower back. Other dynamometer position settings were tailored to each individual to ensure correct participant positioning. The participant’s leg was in line with the dynamometer’s foot support (Figure 1B), with the knee padded and slightly flexed. The axis of rotation of the ankle was at the same level as the axis of the dynamometer foot support. The participants were additionally fixated with straps across the shoulders, pelvis, and distal part of the active limb femur. These fixations were refined through pilot testing and were done to minimize the activation of gluteal and quadriceps muscles.

**FIGURE 2:**
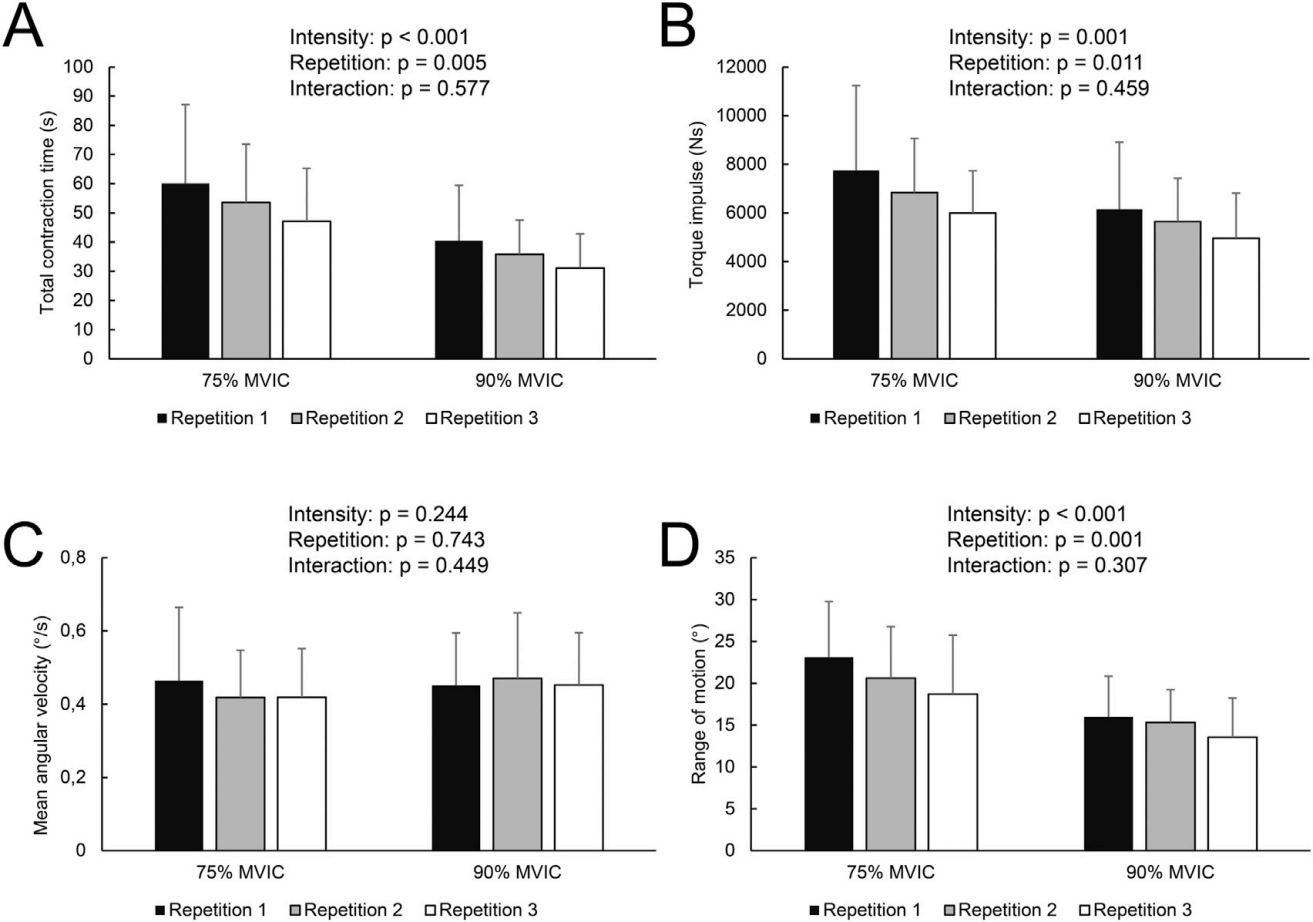
Total time **(A)**, torque impulse **(B)**, mean angular velocity **(C)**, and total range of motion **(D)** across loads and repetitions. p-values indicate main effects of intensity and repetition, as well as their interaction, based on general linear model analysis.

Upon fixating the participant, RoM was determined by asking the participant to place the ankle joint in maximal plantar and dorsal flexion, respectively (Figure 2). Before starting the MVIC measurements, a correction for the effect of gravity was performed in the neutral position. After three warm-up trials for familiarization (first repetition with 50%, second with 75%, and third with 90% of maximum effort), participants performed three 5-s repetitions of MVIC in the neutral ankle position (i.e., 0°), interspersed with 30-s rest intervals. Participants had real-time visual feedback available (time-torque curve) and were verbally encouraged throughout each repetition. After the EQI protocol for each leg (see next section), the procedure was immediately repeated to assess the fatiguing effects.

### 2.4 EQI contractions

The dynamometer was set to the isotonic mode, which allows the resistance to be consistently applied in the same direction (i.e., dorsal flexion in the case of the present study). The average torque was 131.9 ± 29.1 Nm for the 75% MVIC load and 161.3 ± 31.7 Nm for the 90% MVIC load, respectively. Each EQI set consisted of one repetition which started in maximal dorsal flexion. First, participants performed a concentric burst against the direction of resistance (i.e., performing a plantarflexion movement), followed by holding this position and maximally resisting the eccentric contraction by maintaining maximal voluntary plantarflexion torque. Once the dynamometer overcame the participant, the joint moved towards a more dorsiflexed position; consequently, plantar flexors were stretched, which resulted in greater plantarflexion torque capacity. Thus, the participant could maintain the new position for additional time despite prior fatigue. This process, characterized by alternating slow eccentric contractions and isometric holds, continued until maximal dorsal flexion RoM was achieved. The available RoM was the same as determined in MVIC contraction measurements for each participant. While the end RoM was the same as the maximal dorsal flexion RoM, the starting RoM was typically lower than the maximal plantar flexion RoM, as the participants could not push the dynamometer to full RoM against the resistance. Note that in previous studies, EQI contraction typically begun by moving the joint to a position near maximal shortening of the target muscle with examiner’s help. In our study, we operationalized this as the position achieved through maximal concentric effort under load, rather than anatomical end-range, to ensure tolerability and safety. Participants were verbally encouraged throughout the duration of the EQI muscle contraction. The rest period between three same-intensity repetitions was 2 min, and the rest period between testing the other intensity was 5 min. After that, the whole protocol, including a warm-up on the dynamometer, an initial MVIC measurement, three EQI muscle contraction and a final MVIC measurement, was repeated at a different intensity on the other leg.

### 2.5 Data extraction, processing and outcome variables

The data was exported from the dynamometer software (version 9.8.0; CSMi–Computer Sports Medicine Inc., Chicago, United States) to a Microsoft Excel 2013 (Microsoft Corporation, Redmond, Washington, United States) file and then analyzed using MATLAB software (version R2020a; The MathWorks Inc., Natick, Massachusetts, United States). Position (angle plantarflexion/dorsiflexion angle), angular velocity, and torque signals were captured at 100 Hz and processed without prior filtering (Oranchuk et al., 2021). For the MVIC, we considered the peak torque of the best repetition as the only dependent variable.

The start of the EQI muscle contraction was identified at the first local maximum for angle data (i.e., maximal plantar flexion). The end of the EQI contraction was determined when either 1) the end of the RoM was reached or 2) torque values dropped below 50% of the prescribed threshold. All signals were visually inspected for artefacts that could affect the determination of EQI muscle contraction onset and offset, with manual corrections made where needed (n = 3 contractions). To mitigate the risk of type 1 error rates and associated false discovery rates, our analysis primarily concentrated on four outcome variables: 1) the total duration of the EQI muscle contraction (s), 2) the total torque impulse (area under torque-time curve determined with the trapezoid integration) (Nm⋅s); 3) the mean angular velocity (°/s) and 4) total RoM (°). Although the final angle (end-RoM) was expected to be fairly consistent among repetitions, the fatigue of prior repetitions could cause the participant to reach a smaller initial plantar flexion with the concentric contraction. Consequently, including RoM as a dependent variable in the study was considered crucial to obtain a holistic picture of the differences among intensities and subsequent repetitions.

### 2.6 Statistical analysis

The data are presented as means ± standard deviations. The normality of the data distributions for all variables was verified with the Shapiro-Wilk test and visual inspection of histograms and Q-Q plots. Preliminary analysis indicated no differences between left and right legs (F = 0.262–0.455; p = 0.411–0.615), nor any leg × sex interactions (F = 0.001–0.321; p = 0.771–0.985). Therefore, the side factor (left, right) was not further considered as a factor in the main analyses. MVIC torque was analyzed with a general linear model which included time (before and after EQI muscle action) and load (75% and 90% MVIC) as within-subject factors and sex (men, women) as a between-subject factor. For EQI muscle action, a general linear model with load (75% and 90% MVIC) and repetition (1st, 2nd, 3rd) as within-subjects factors and sex as between-subject factors was applied. Mauchly’s test was used to assess sphericity and the Greenhouse-Geisser correction was applied to adjust for any violations of sphericity. Bonferroni-corrected *post hoc* testing was used to assess pairwise differences among repetitions. Effect sizes were calculated as partial eta-squared (η_p_^2^) and were considered to indicate no effect (<0.01), a small effect (0.01–0.06), a medium effect (0.06–0.14), and a large effect (>0.14). In case of significant 2-way or 3-way interactions, estimated marginal means with 95% confidence intervals (CI) and *post hoc* 1-way analyses of variance were conducted. The threshold for statistical significance was set at α < 0.05 and all analyses were carried out in SPSS statistical software (version 25.0, IBM, United States).

## 3 Results

The demographic details are shown in Table 1. Men were statistically significantly heavier and taller (p < 0.001) and produced larger maximal voluntary isometric contraction (MVIC) torque (p = 0.011 and p = 0.002 for left and right leg, respectively), while there were no differences in age (p = 0.455).

**TABLE 1:** Basic participants’ characteristics.

Variable	Men		Women		Difference		
	Mean	SD	Mean	SD	T-value	Sig. (p)	Effect size
Age (years)	25.1	3.1	24.1	3.3	0.76	0.455	0.34
Body height (cm)	183.2	4.5	169.9	5.8	5.77	<0.001***	2.58
Body mass (kg)	84.0	6.9	66.5	5.4	6.32	<0.001***	2.82
MVIC torque - Left leg (Nm)	202.7	38.6	159.1	29.7	2.77	0.011*	1.27
MVIC torque - Right leg (Nm)	199.0	29.7	155.1	25.0	3.52	0.002**	1.60

MVIC, Maximal voluntary isometric contraction; SD, standard deviation.

Effect size is reported as Cohen’s d. *p < 0.05, **p < 0.01, ***p < 0.001.

### 3.1 Maximal voluntary contraction

The analysis indicated no statistically significant effect of EQI protocol on absolute MVIC torque (main effect of time: p = 0.636) (Table 2). No statistically significant main effect of time was observed between 75% and 90% MVIC loads (p = 0.589), and there was also no load × time interaction (p = 0.314). As expected from the baseline sex comparisons (Table 1), there was a large sex effect (F = 10.3; p = 0.005; η_p_^2^ = 0.37). The remaining interaction effects analyzed in the general linear model (not shown in Table 2) were also not statistically significant (load × sex: p = 0.897; time × sex: p = 0.168; time × sex × load: p = 0.850).

**TABLE 2:** MVIC values before and after EQI muscle actions at different loads in men and women.

Load	Time	Men		Women		Statistical analysis			
		Mean	SD	Mean	SD	Factor	F	p	η_p_^2^
75% MVIC	Pre	199.6	41.2	161.5	33.9	Load	0.392	0.539	0.02
	Post	199.4	38.1	154.6	27.7	Time	0.233	0.636	0.01
90% MVIC	Pre	195.0	27.8	154.6	26.7	Sex	10.38	0.005**	0.37
	Post	199.0	35.9	153.9	23.5	Load × time	1.07	0.314	0.06

MVIC, maximal voluntary isometric contraction; SD, standard deviation.

*p < 0.05, **p < 0.01, ***p < 0.001.

### 3.2 EQI muscle actions

For EQI muscle contraction time, there was no main effect of sex (p = 0.433), load × repetition interaction (p = 0.577), nor load × sex (p = 0.526), repetition × sex (p = 0.588), and repetition × sex × load (p = 0.342) interactions. There was a large and statistically significant effect of load (F = 29.2; p < 0.001; η_p_^2^ = 0.60), as well as repetition (F = 8.5; p = 0.005; η_p_^2^ = 0.31). Specifically, the total time was shorter with every subsequent repetition and during 90% compared to 75% MVIC load (Figure 2A). Post-hoc tests demonstrated that Repetition 3 was shorter compared to Repetition 1 (p = 0.013) and Repetition 2 (p = 0.003), while there was no statistically significant difference between Repetition 1 and 2 (p = 0.208).

Torque impulse also showed statistically significant and large effects of load (F = 17.2; p = 0.001; η_p_^2^ = 0.48) and repetition (F = 7.0; p = 0.011; η_p_^2^ = 0.27), without statistically significant differences between sexes (p = 0.331) and any statistically significant interactions (p = 0.251–0.779). Torque impulse was larger at 75% MVIC compared to 90% MVIC and was decreasing with subsequent repetitions (Figure 2B); similar to total contraction time, *post hoc* tests showed that Repetition 3 had lower torque impulse compared to Repetition 1 (p = 0.024) and Repetition 2 (p = 0.001), while there was no statistically significant difference between Repetition 1 and 2 (p = 0.335).

For mean angular velocity (Figure 2C), there was no effect of load (p = 0.244), repetition (p = 0.743), and sex (p = 0.942). In addition, there were no statistically significant 2-way or 3-way interactions (p = 0.154–0.893).

For total RoM, there was a large and statistically significant effect of load (F = 25.8; p < 0.001; η_p_^2^ = 0.58) and repetition (F = 9.72; p = 0.001; η_p_^2^ = 0.34). Total RoM was larger in 75% MVIC compared to 90% MVIC and decreased with subsequent repetitions (Figure 2D). Post-hoc tests showed that Repetition 3 was characterized by a smaller RoM compared to Repetition 1 (p = 0.005) and Repetition 2 (p = 0.015), while there was no statistically significant difference between Repetition 1 and 2 (p = 0.174). There was no load × repetition interaction (p = 0.307). There was a statistically significant effect of sex (F = 5.35; p = 0.032; η_p_^2^ = 0.22) and a statistically significant sex × load interaction (F = 4.65; p = 0.044; η_p_^2^ = 0.20). There was no repetition × sex (p = 0.794) and no repetition × sex × load (p = 0.888) interactions. Additional analysis of estimated marginal means (pooled across repetitions) showed that women had larger RoM compared to men at 75% MVIC (23.4° (95% CI = 20.5–26.4°) compared to 15.6° (95% CI = 13.1–18.1°); p = 0.009), but not at 90% MVIC (15.6° (95% CI = 13.1–18.1°) compared to 14.1° (95% CI = 11.2–16.9); p = 0.413).

Further analysis was done to discern whether the starting angle (i.e., the plantar flexion angle to which the participants were able to push concentrically before starting the EQI muscle action) or the final angle was driving the differences in RoM. Starting angle displayed only statistically significant effects of load (F = 13.1; p = 0.002; η_p_^2^ = 0.40) and repetition (F = 14.5; p < 0.001; η_p_^2^ = 0.40), with no sex effects (p = 0.784) nor any interaction (p = 0.101–0.530). Additional analysis of values between loads showed that the starting angle was statistically significantly larger (i.e., more plantaflexed) for the 75% MVIC load compared to 90% MVIC (mean difference: repetition 1: +6.1 ± 7.4°, p = 0.001; repetition 2: 3.7 ± 4.7°, p = 0.002; repetition 3: 3.6 ± 6.2°, p = 0.017). Conversely, the final angle did not statistically significantly differ among intensities (p = 0.137) nor across repetitions (p = 0.441). This was expected, as the participants were required to perform the EQI muscle contraction to the maximal available RoM. However, the final angle showed a statistically significant effect of sex (F = 9.66; p = 0.006; η_p_^2^ = 34), as well as a statistically significant sex × load interaction (F = 5.71; p = 0.027; η_p_^2^ = 0.23). There were no other statistically significant interactions (p = 0.301–0.631). Additional analysis of estimated marginal means (pooled across repetitions) showed that women had larger final angle than men at 75% MVIC (15.8° (95% CI = 13.7–17.9°) compared to 9.8° (95% CI = 7.4–12.2°); p = 0.001), but not at 90% MVIC (12.9° (95% CI = 10.8–15.1°) compared to 10.4° (95% CI = 7.9–12.8°); p = 0.128). Overall, this suggests that different starting angles caused the differences in RoM among loads and repetitions, while a different final angle was the primary cause of sex differences and sex × load interactions.

## 4 Discussion

In this study, we explored the effects of EQI muscle actions on ankle plantar flexors at two high loads (75% and 90% MVIC). No significant decrease in MVIC torque following three EQI setss at 75% or 90% MVIC was observed. We observed a statistically significant larger total contraction time, total torque impulse, and RoM at 75% compared to 90% MVIC intensity, suggesting the superiority of the 75% MVIC load for imparting a large cumulative load to the ankle plantar flexors.

One of the most interesting findings was that the execution of three repetitions of EQI (regardless of load) did not cause a decrease of MVIC torque while comparing pre- to post-measurement (Table 2). To our knowledge, no studies have been conducted to determine the effects of a single-bout eccentric and/or isometric ankle plantar flexor exercise on MVIC torque, while the execution of a single bout isotonic contraction (calf raises) resulted in decreased ankle plantar flexor MVIC (Obst et al., 2013). Meanwhile, research suggests that both knee extensors and knee flexors experience a decrease in MVIC after performing eccentric contraction (Emery et al., 1994) and isometric contraction (Massamba et al., 2022). Oranchuk et al. (2021) found out that the knee extensor MVIC decrease after performing the EQI contractions is lower than in the case of an isolated eccentric contraction (when the total torque impulse is equalized). Considering the limited research available, it seems reasonable to explore the impact of ankle plantar flexor fatigue on MVIC torque post-EQI, in comparison with equivalent extensive eccentric contractions, to ascertain if EQI exercise result in less fatigue than alternative contraction modalities, comparable to what occurs with knee extensors (Oranchuk et al., 2021).

As expected, we observed that total torque impulse and time under tension decreased across subsequent repetitions of EQI muscle actions. Lower total torque impulse and time under tension with subsequent repetitions were also observed by Oranchuk et al. (2021) for knee extensors. We also found out that both, total torque impulse and time under tension were greater in the case of 75% load compared to 90% load (Figure 2B). The fact that a higher total torque impulse was observed at the 75% load is likely mostly related to a longer time under tension at 75% load (Figure 2A). This means that although the load was lower, longer contraction enabled a larger impulse to accumulate. We also found out that the mean angular velocity during EQI muscle actions was not statistically significantly different across loads and across repetitions (Figure 2C), suggesting that differences in total time are paralleled with differences in total RoM, while the velocity is relatively constant. Meanwhile, RoM decreased with subsequent repetitions in both intensities and was lower while executing EQI at 90% load (Figure 2D). RoM during EQI contraction was also greater in women compared to men (Soucie et al., 2011), however, this difference was due to a greater end-RoM (i.e., larger dorsiflexion RoM in women) and not the starting RoM achieved with concentric contraction before EQI muscle action.

In our study, we also compared the contraction rates between the two intensities. We found that the angular velocity of the EQI contraction of the plantar flexors of the ankle was markedly lower than that reported for the elbow flexors, notwithstanding that in the mentioned study the subjects performed the protocol at an intensity equal to 50% of the previously measured MVIC torque, while the load of our study was defined at 75 or 90% of participant’s MVIC (Henderson et al., 2023). Although the range of motion was greater in elbow flexors compared to both intensities in our study and elbow flexors experienced lower load intensity, the differences can be interpreted in line with the findings of histological studies showing that m. triceps surae is a significantly slower muscle compared to m. biceps brachii, while ankle plantar flexors (especially m. triceps surae) are a postural muscles, while the latter is used mainly in the locomotion phase and in slightly more rapid and explosive movements (Dahmane, 2005).

### 4.1 Potential practical application

Based on the previous studies and our findings, we believe that EQI exercise could have potential for enhancing exercise-based programs. While the EQI protocols investigated in this study (75% and 90% of MVIC torque) can be classified as moderate- and high-load conditions, our findings suggest that both protocols may be appropriate for regularly active individuals. However, the 75% MVIC protocol appears more favorable, as it resulted in a greater total torque impulse and a larger range of motion, indicating a potentially more effective mechanical stimulus despite the lower load. The findings of our study could be applied to a practical situation in individuals who do not have access to an isokinetic dynamometer to perform finely controlled EQI muscle actions For instance, an alternative approach using a Smith machine for unilateral EQI muscle actions of the ankle plantar flexors could be used. After determining the unilateral one repetition maximum (1 RM), participants would calculate 75% of the determined 1 RM load and perform a bilateral concentric contraction of the ankle plantar flexors, immediately followed by an EQI contraction through the full RoM. The Smith machine would facilitate a safer and as optimized as possible execution of this protocol outside of a laboratory setting. However, future studies are warranted to investigate the biomechanical characteristics of EQI muscle actions performed on a Smith machine.

### 4.2 Potential clinical application

This detailed examination of EQI muscle contraction torque, time under tension, velocity, and RoM can serve as a potential starting point to explore the efficacy of EQI exercsie for tendon rehabilitation. In case of the Achilles tendon, injuries are most frequently referred to Achilles tendinopathy (Sharma and Maffulli, 2006). The latter is characterized by swelling, pain, and impaired performance of the Achilles tendon (Alfredson, 2003; Maffulli et al., 1998; van Dijk et al., 2011). The field of conservative management of Achilles tendinopathy is relatively well-researched. While some protocols now incorporate well-defined, objective loading criteria (e.g., Radovanović et al., 2022), the main criticism of most exercise-therapeutic protocols (e.g., Alfredson protocol, HSR protocol, Silbernagel combined protocol, etc.) is the imprecise determination of the load that needs to be added at later stages, where increasing the load is mostly based on the subjective sensation of pain level, which should be around 5 on a 10-point scale, and subjective feeling that the patient will be able to perform a certain number of quality repetitions (Luan et al., 2019). Since EQI exercise is based on the well-defined load (even in case of Smith machine variant) and because 70%–75% MVIC is considered sufficient to induce an Achilles tendon strain of approximately 4.0%–4.5% (McCrum et al., 2018; Wiesinger et al., 2015), both intensities can potentially be considered adequate to promote tendon adaptations such as tendon cross-sectional area, stiffness and Young’s modulus increase (Arampatzis et al., 2007; Bohm et al., 2015; Wiesinger et al., 2015). However, it is important to note that not only the magnitude of strain, but also its duration, and the strain-time integral is potentially critical in triggering tendon adaptation. Before providing a firmer conclusion on the potential use of the EQI exercise for rehabilitation, more detailed studies investigating the morphological, biomechanical and fatigue effects after the implementation of at least 12-week isometric, eccentric or EQI protocol should be executed. In addition, studies to determine the most appropriate intensity and the most appropriate rules for increasing loads within EQI protocol should be carried out prior to a study examining the effects on specific pathologies.

### 4.3 Limitations

Some limitations of our study must be acknowledged. First, because the study participants were young and regularly physically active, their results are not directly transferable to the general, older, and less physically active population. Furthermore, we found out that neither the load at 75% nor at 90% of MVIC caused statistically significant effects on post-protocol MVIC torque. We believe that these results are since, as mentioned above, the participants were regularly physically active and only one series of three EQI repetitions for each intensity was performed. Therefore, the fatiguing effects of a more extensive EQI protocol remain to be explored. An additional limitation of the study is the non-standardization of knee position between subjects. The knee was slightly flexed due to discomfort when the protocol was performed and this level was not precisely controlled, which may have influenced the results. A further limitation is that EQI contractions began at the end of active concentric plantarflexion rather than full anatomical plantarflexion, to reduce the risk of cramping. While this approach improved safety, it may affect comparability with previous protocols starting from a more shortened muscle position.

## 5 Conclusion

During the comparison of the fatigue effects and biomechanical characteristics of ankle plantar flexors between the execution of EQI muscle actions at two different loads (at 75% and 90% MVIC), we found that neither 75% load nor 90% load caused a statistically significant decrease on MVIC torque when comparing MVIC torque pre- and post-protocol. The mean angular velocity was also similar between the two intensities. Participants achieved longer contraction time, higher total torque impulse and greater RoM during the execution of EQI muscle actions at 75% MVIC compared to 90% MVIC. These results indicate that EQI muscle actions at 75% MVIC enable the application of greater cumulative load within the same number of repetitions.

## Data Availability

Raw data used for statistics, and raw signals for individual subject are freely available on Zenodo (https://doi.org/10.5281/zenodo.15679911).

## References

[B1] Alfredson H. (2003). Chronic midportion achilles tendinopathy: an update on research and treatment. Clin. Sports Med. 22 (4), 727–741. 10.1016/S0278-5919(03)00010-3 14560544

[B2] Alfredson H. Pietila T. Jonsson P. Lorentzon R. (1998). Heavy-load eccentric calf muscle training for the/Treatment of chronic achilles tendinosis. Am. J. Sports Med. 26 (3), 360–366. 10.1177/03635465980260030301 9617396

[B3] Arampatzis A. Karamanidis K. Albracht K. (2007). Adaptational responses of the human achilles tendon by modulation of the applied cyclic strain magnitude. J. Exp. Biol. 210 (15), 2743–2753. 10.1242/jeb.003814 17644689

[B4] Arnoczky S. P. Lavagnino M. Egerbacher M. (2007). The mechanobiological aetiopathogenesis of tendinopathy: is it the over-stimulation or the under-stimulation of tendon cells? Int. J. Exp. Pathology 88 (4), 217–226. 10.1111/j.1365-2613.2007.00548.x PMC2517314 17696902

[B5] Bohm S. Mersmann F. Arampatzis A. (2015). Human tendon adaptation in response to mechanical loading: a systematic review and meta-analysis of exercise intervention studies on healthy adults. Sports Med. 1 (1), 7. 10.1186/s40798-015-0009-9 PMC4532714 27747846

[B34] Carvalho L. Junior R. M. Barreira J. Schoenfeld B. J. Orazem J. Barroso R. (2022). Muscle hypertrophy and strength gains after resistance training with different volume-matched loads: a systematic review and meta-analysis. Appl. Physiol. Nutr. 47 (4), 357–368. 10.1139/apnm-2021-0515 35015560

[B6] Dahmane R. G. Djordjevic S. Simunic B. Valencic V. (2005). Spatial fiber type distribution in normal human muscle histochemical and tensiomyographical evaluation. J. Biomech. 38, 2451–2459. 10.1016/j.jbiomech.2004.10.020 16214493

[B35] Emery L. Sitler M. Ryan J. (1994). Mode of action and angular velocity fatigue response of the hamstrings and quadriceps. Isokinetics and exercise science, 4(3), 91–95. 10.3233/IES-1994-4301

[B7] Grävare-Silbernagel K. Thomee P. Karlsson J. Thomee R. (2001). Eccentric overload training for patients with chronic achilles tendon pain--a randomised controlled study. Scand. J. Med. Sci. Sports 11, 197–206. 10.1034/j.1600-0838.2001.110402.x 11476424

[B8] Habets B. van Cingel R. E. H. (2015). Eccentric exercise training in chronic mid-portion achilles tendinopathy: a systematic review on different protocols. Scand. J. Med. Sci. Sports 25 (1), 3–15. 10.1111/sms.12208 24650048

[B9] Head J. Mallows A. Debenham J. Travers M. J. Allen L. (2019). The efficacy of loading programmes for improving patient-reported outcomes in chronic midportion achilles tendinopathy: a systematic review. Musculoskelet. Care 17 (4), 283–299. 10.1002/msc.1428 31763774

[B10] Henderson Z. J. Wang S. Cornish S. M. Scribbans T. D. (2023). Exploring the acute muscle fatigue response in resistance trained individuals during eccentric quasi-isometric elbow Flexions—A cross-sectional comparison of repetition and sex. Sport Rx IV Prepr. 2003, 1–23. 10.1080/14763141.2023.2269543 37921046

[B11] Jenkins N. D. M. Housh T. J. Bergstrom H. C. Cochrane K. C. Hill E. C. Smith C. M. (2015). Muscle activation during three sets to failure at 80 vs. 30 % 1RM resistance exercise. Eur. J. Appl. Physiology 115 (11), 2335–2347. 10.1007/s00421-015-3214-9 26159316

[B12] Ličen U. Oranchuk D. J. Kozinc Ž. (2024). Exploring the biomechanics and fatigue patterns of eccentric quasi-isometric muscle actions in the knee extensors and flexors. Eur. J. Appl. Physiology 124, 3409–3419. 10.1007/s00421-024-05544-w 38953975

[B13] Luan X. Tian X. Zhang H. Huang R. Li N. Chen P. (2019). Exercise as a prescription for patients with various diseases. J. Sport Health Sci. 8 (5), 422–441. 10.1016/j.jshs.2019.04.002 31534817 PMC6742679

[B14] Maffulli N. Khan K. M. Puddu G. (1998). Overuse tendon conditions: time to change a confusing terminology. Arthroscopy 14 (8), 840–843. 10.1016/S0749-8063(98)70021-0 9848596

[B15] Magnusson S. P. Kjaer M. (2019). The impact of loading, unloading, ageing and injury on the human tendon. J. Physiology 597 (5), 1283–1298. 10.1113/JP275450 PMC6395417 29920664

[B16] Malliaras P. Barton C. J. Reeves N. D. Langberg H. (2013). Achilles and patellar tendinopathy loading programmes: a systematic review comparing clinical outcomes and identifying potential mechanisms for effectiveness. Sports Med. 43 (4), 267–286. 10.1007/s40279-013-0019-z 23494258

[B36] Massamba A. Hucteau E. Mallard J. Ducrocq G. P. Favret F. Hureau T. J. (2022). Exercise-induced fatigue in hamstring versus quadriceps muscles and consequences on the torque–duration relationship in men. Med. Sci. Sports. Exerc., 54 (12), 2099–2108. 10.1249/MSS.0000000000003007 35868018

[B17] McCrum C. Leow P. Epro G. König M. Meijer K. Karamanidis K. (2018). Alterations in leg extensor muscle-tendon unit biomechanical properties with ageing and mechanical loading. Front. Physiology 9 (FEB), 150–157. 10.3389/fphys.2018.00150 PMC5835978 29541035

[B18] Merza E. Pearson S. Lichtwark G. Ollason M. Malliaras P. (2021). Immediate and long-term effects of mechanical loading on achilles tendon volume: a systematic review and meta-analysis. J. Biomechanics 118, 110289. 10.1016/j.jbiomech.2021.110289 33556887

[B19] Murphy M. C. Travers M. J. Chivers P. Debenham J. R. Docking S. I. Rio E. K. (2019). Efficacy of heavy eccentric calf training for treating mid-portion achilles tendinopathy: a systematic review and meta-analysis. Br. J. Sports Med. 53 (17), 1070–1077. 10.1136/bjsports-2018-099934 30636702

[B20] Obst S. J. Barrett R. S. Newsham-West R. (2013). Immediate effect of exercise on achilles tendon properties: systematic review. Med. Sci. Sports Exerc. 45 (8), 1534–1544. 10.1249/MSS.0b013e318289d821 23439426

[B21] Oranchuk D. J. Diewald S. N. McGrath J. W. Nelson A. R. Storey A. G. Cronin J. B. (2021). Kinetic and kinematic profile of eccentric quasi-isometric loading. Sports Biomech. 00 (00), 758–771. 10.1080/14763141.2021.1890198 33666143

[B22] Oranchuk D. J. Nelson A. R. Storey A. G. Diewald S. N. Cronin J. B. (2021). Short-term neuromuscular, morphological, and architectural responses to eccentric quasi-isometric muscle actions. Eur. J. Appl. Physiology 121 (1), 141–158. 10.1007/s00421-020-04512-4 32995961

[B23] Oranchuk D. J. Storey A. G. Nelson A. R. Cronin J. B. (2019). Scientific basis for eccentric quasi-isometric resistance training: a narrative review. J. Strength Cond. Res. 33 (Issue 10), 2846–2859. 10.1519/JSC.0000000000003291 31361732

[B24] Rabello L. M. Van Den Akker-Scheek I. Brink M. S. Maas M. Diercks R. L. Zwerver J. (2020). Association between clinical and imaging outcomes after therapeutic loading exercise in patients diagnosed with achilles or patellar tendinopathy at short- and long-term follow-up: a systematic review. Clin. J. Sport Med. 30 (4), 390–403. 10.1097/JSM.0000000000000624 29952842

[B25] Radovanović G. Bohm S. Peper K. K. Arampatzis A. Legerlotz K. (2022). Evidence-based high-loading tendon exercise for 12 weeks leads to increased tendon stiffness and cross-sectional area in achilles tendinopathy: a controlled clinical trial. Sports Med. - Open 8 (1), 149. 10.1186/s40798-022-00545-5 36538166 PMC9768072

[B26] Schoenfeld B. J. Contreras B. Willardson J. M. Fontana F. Tiryaki-Sonmez G. (2014). Muscle activation during low-*versus* high-load resistance training in well-trained men. Eur. J. Appl. Physiology 114 (12), 2491–2497. 10.1007/s00421-014-2976-9 25113097

[B27] Schoenfeld B. J. Grgic J. Ogborn D. Krieger J. W. (2017). Strength and hypertrophy adaptations between Low-vs. high-load resistance training: a systematic review and meta-analysis. J. Strength Cond. Res. 31 (12), 3508–3523. 10.1519/JSC.0000000000002200 28834797

[B28] Sharma P. Maffulli N. (2006). Understanding and managing achilles tendinopathy. Br. J. Hosp. Med. 67 (2), 64–67. 10.12968/hmed.2006.67.2.20463 16498904

[B29] Soucie J. M. Wang C. Forsyth A. Funk S. Denny M. Roach K. E. (2011). Range of motion measurements: reference values and a database for comparison studies. Haemophilia 17 (3), 500–507. 10.1111/j.1365-2516.2010.02399.x 21070485

[B30] van Dijk C. N. van Sterkenburg M. N. Wiegerinck J. I. Karlsson J. Maffulli N. (2011). Terminology for achilles tendon related disorders. Knee Surg. Sports Traumatol. Arthrosc. 19 (5), 835–841. 10.1007/s00167-010-1374-z 21222102 PMC3076576

[B31] Wiegerinck J. I. Kerkhoffs G. M. van Sterkenburg M. N. Sierevelt I. N. van Dijk C. N. (2013). Treatment for insertional achilles tendinopathy: a systematic review. Knee Surg. Sports Traumatol. Arthrosc. 21 (6), 1345–1355. 10.1007/s00167-012-2219-8 23052113

[B32] Wiesinger H. P. Kösters A. Müller E. Seynnes O. R. (2015). Effects of increased loading on *in vivo* tendon properties: a systematic review. Med. Sci. Sports Exerc. 47 (9), 1885–1895. 10.1249/MSS.0000000000000603 25563908 PMC4535734

[B33] Young J. L. Press J. M. (1994). The physiologic basis of sports rehabilitation. Phys. Med. Rehabilitation Clin. N. Am. 5 (1), 9–36. 10.1016/S1047-9651(18)30536-9

